# Nesfatin-1 Stimulates Fatty-Acid Oxidation by Activating AMP-Activated Protein Kinase in STZ-Induced Type 2 Diabetic Mice

**DOI:** 10.1371/journal.pone.0083397

**Published:** 2013-12-31

**Authors:** Jing Dong, Huan Xu, Huan Xu, Peng-fei Wang, Gui-ju Cai, Hai-feng Song, Chang-chen Wang, Zhao-tong Dong, Yan-jiao Ju, Zheng-yao Jiang

**Affiliations:** 1 Physiology Department of Medical College, Qingdao University, Qingdao, China; 2 Special Medicine Department of Medical College, Qingdao University, Qingdao, China; 3 Class 2, Grade 2009, Medical College, Qingdao University, Qingdao, China; 4 Grade 2009, Medical College, Qingdao University, Qingdao, China; 5 Grade 2008, Medical College, Qingdao University, Qingdao, China; 6 Grade 2010, Medical College, Qingdao University, Qingdao, China; Medical University Innsbruck, Austria

## Abstract

Nesfatin-1 is an anorexigenic peptide involved in energy homeostasis. Recently, nesfatin-1 was reported to decrease blood glucose level and improve insulin sensitivity in high-fat diet-fed rats. However, little information is known about the influence of nesfatin-1 on lipid metabolism either in physiological or diabetic condition. This study undertook whether nesfatin-1 was involved in the pathophysiology in Streptozotocin-induced type 2 diabetic mice (T2DM), which was induced by a combination of high-calorie diet and two low-doses Streptozotocin. We observed that plasma nesfatin-1 was significantly increased while expression of nesfatin-1 neurons were decreased in hypothalamus in diabetes group compared to only high-calorie diet control group; intravenous injection of nesfatin-1 decreased 0–1h, 0–2h, 0–3h cumulative food intake in T2DM, but 0–24h total food intake had no difference between groups. Body weight and plasma FFA were normalized after nesfatin-1(10 µg/Kg) administration for 6 days. These results suggested that nesfatin-1 improved lipid disorder in T2DM. It was found that blood glucose and insulin resistance coefficient decreased with treatment of nesfatin-1 (both in 1 µg/Kg and 10 µg/Kg doses) in diabetes mice. For further understanding the role of nesfatin-1 on lipid metabolism, we detected p-AMPK and p-ACC of skeletal muscle in T2DM using western blotting. The expression of p-AMPK and p-ACC increased when nesfatin-1 was given with doses 1 µg/Kg but not in doses 10 µg/Kg. Taken together, nesfatin-1 participated in the development of T2DM and stimulated free fatty acid utilization via AMPK-ACC pathway in skeletal muscle in T2DM.

## Introduction

Nesfatin-1 has been identified as a leptin-independent anorectic peptide, which processed from nucleobindin 2 (NUCB2) [1]. Both central and peripheral injections of nesfatin-1 caused a significant reduction of food intake and body weight in rodents [2]. The permeation of nesfatin-1 was a non-saturable process in either the blood-to-brain or brain-to-blood direction [3], [4]. Central nesfatin-1 activated oxytocin neurons in the PVN and project to POMC neurons in the NTS, causing melanocortin dependent anorexia [5]. In periphery, NUCB2/nesfatin-1 positive cells were located mainly in gastric mucosa, pancreatic islet β-cell [6] and adipose tissue [7]. Nesfatin-1 had the function of anti-hyperglycemia in a insulin dependent manner [8]. In addition, nesfatin-1 enhanced glucose-stimulated insulin secretion in vivo through the Ca^2+^ channels [9]. Continuous peripheral infusion of nesfatin-1 improved insulin sensitivity in rats. Moreover, it was also found to increase spontaneous physical activity, whole-body fat oxidation in rats [2]. Collectively, these data suggested that nesfatin-1 participated in energy homeostasis.

In T2DM, plasma nesfatin-1 concentration and tissue expression were changed compared with the control animal, which may indicate nesfatin-1 participated in diabetes development. Our previous study firstly reported that plasma nesfatin-1 decreased in treated T2DM patients [10]. However, there was increased levels of plasma nesfatin-1 in newly diagnosed T2DM patients [11]. Moreover, pancreatic islet NUCB2 mRNA decreased in T2DM patients [12] and protein levels of NUCB2 also decreased in Goto-Kakizaki rats [13]. In this study, we observed the expression and concentration of nesfatin-1 in T2DM in order to reveal the relationship between nesfatin-1 and T2DM and try to detect a new target for the treatment of T2DM.

Most T2DM patients could not produce enough insulin or their cells ignored insulin. T2DM patients were characterized with disorders of fatty-acid metabolism. Increased lipid stores in non-adipose tissues such as muscle were linked to functional impairments, called lipotoxicity [14]. Impaired fuel metabolism in diabetes may lead to chronic accumulation of lipid oxidative metabolites within blood or tissues, which played a key role in insulin resistance and insulin secretion [15]. Nesfatin-1, as described above, was a metabolic regulator. Interestingly, Infusion of nesfatin-1 into the third ventricle could inhibit hepatic glucose production and promote glucose uptake significantly [16]. In addition, central nesfatin-1 could activate insulin receptor (InsR)/insulin receptor substrate-1 (IRS-1)/AMP-dependent protein kinase (AMPK)/Akt kinase (Akt)/target of rapamycin complex (TORC) 2 phosphorylation in skeletal muscle [16], while intravenous injection of nesfatin-1 significantly reduced blood glucose in hyperglycemic db/db mice [8]. However, little is known about the influence of peripheral nesfatin-1 on lipid metabolism either in physiological condition or in T2DM.

Skeletal muscle was responsible for the majority of insulin- mediated glucose uptake in peripheral tissue because of its large mass. Fasting plasma free fatty acid (FFA) increased in T2DM and chronic exposure of FFA has been shown to lead to skeletal muscle insulin resistance [15]. AMP-activated protein kinase (AMPK), a prominent metabolic enzyme, acted to improve insulin sensitivity and the whole body energy balance [17]. Many antidiabetic medications, such as metformin and the thiazolidinediones, were reported to activate AMPK [18]. In skeletal muscle, the main isoform was α-AMPK [14]. Phosphorylated AMPK (p-AMPK) phosphorylated acetyl CoA-carboxylase (ACC) and then leaded to the decrease of malonyl-CoA levels, thus stimulating mitochondrial carnitine palmitoyltransferase 1 (CPT1) and promoting FFA oxidation [17].

This study aimed to explore whether peripheral nesfatin-1 was involved in the pathophysiology in streptozotocin (STZ)-induced T2DM mice and, if so, how nesfatin-1 ameliorated disordered lipid metabolism, whether AMPK in skeletal muscle was a target of peripheral nesfatin-1.

## Materials and Methods

### 1. Animals

Male Kunming SPF mice (Institute of Drug Control of Qingdao, China) were used. All animals had free access to food and water in a 12h light/dark cycle and housed in temperature controlled animal room. They were fed with high-fat diet (59% basic mice feed, 20% sugar, 18% lard, and 3% egg yolk). The protocols used for handling the mice were approved by Qingdao University Animal Care and Use Committee and in accordance with the guidelines set by the National Institutes of Health Guide for Care and Use of Laboratory [19]. At the age of 6 and 9 weeks, mice were rendered twice low-dose streptozocin (100 mg/kg, i.p.) [19]–[21]. Mice with fasting blood glucose levels above 12 mmol/L and higher insulin levels were considered type 2 diabetic mice.

The animals were randomly divided into four groups: HFDN group (high fat diet+NS, 0.9% normal saline), DMN group (DM +NS), DML group (DM+low-dose nesfatin-1, 1 µg/Kg) and DMH group (DM+high-dose nesfatin-1, 10 µg/Kg). Mice in HFDN group were rendered high-fat diet without streptozocin (STZ) injection. At the 10-week age, both HFDN group and DMN group were intravenously administrated with normal saline once a day (7:00 pm), while DML and DMH group were offered nesfatin-1 (1 µg/Kg or 10 µg/Kg) once a day (7:00 pm). Injections were started in the evening (day 1–6), with the last injection in the morning (day 7). Animals were sacrificed 2h after the last injection, with food withdrawal 12h before death. Blood samples were obtained from the orbital sinus and stored in –20°C while other samples in –80°C until use.

### 2. Body weight and glucose measurement

The blood glucose was measured by tail vain blood with a glucometer (BRAUN Omnitest®EZ). As insulin resistance was not the primary interest, it was assessed by the formula: HOMA IR = fasting plasma insulin (µU/ml)×fasting plasma glucose (mmol/l)/22.5. Then the scores were log-transferred (log-transformed HOMA) to determine a much stronger linear correlation with glucose [22]–[24].

Body weights were measured with an electric balance (Mettler Toledo, PL1501-S, Shanghai, China).

### 3. Food intake

Food intake was detected by measuring the weight of food containers with electronic precision scales (Feeding and Activity Analyser 47552-002, Ugo Basile, Italy). The outputs were continuously monitored by Data Acquisition software 51800 (Feed-Drink Monitoring System, Ugo Basile, Italy)

### 4. Biochemical analysis

Nesfatin-1 and insulin levels were determined by mouse ultrasensitive ELISA Kit (R&D Systems and Wuhan ColorfulGene biological technology, China).The free fatty acid (FFA) levels were measured by an ultrasensitive assay kit for free fatty acid (Applygen Technologies Inc. Beijing, China).

### 5. Western blot analysis

Muscle strips were homogenized in ice cold standard RIPA buffer with protease and phosphatase inhibitors Cocktail (Sigma) and centrifuged at 4**°**C. We used primary rabbit antibodies for phosphorylated-AMPK (Thr172, Cell Signaling) and total-AMPK (Cell Signaling) or phosphorylated-ACC (Ser79, Cell Signaling) and total-ACC (Cell Signaling) in TBST at a dilution of 1∶2000. The secondary antibody was peroxidase-conjugated goat anti-rabbit IgG (Beijing Zhongshan Jinqiao Biotec) used at a 1∶8000 dilution. The intensity of bands was analyzed using Quantity One software (Bio-Rad).

### 6. Immunofluorescence

Tissues were fixed in 4% paraformaldehyde, and then dehydrated in 30% sucrose. Both of the fixation and dehydration were followed by a wash in PBS for three times. Brains were frozen and sectioned at 20 µm thickness by a cryostat microtome (CM1900, Leica, Germany). After a microwave repair for the antigen, blocking solution (1%BSA+0.1%Tween-20 in PBS) was added. Primary antibody (Phoenix Pharmaceuticals. Inc.) diluted in blocking solution at a recommended ratio of 1:1000 was added. Then incubated them overnight at 4°C. Incubated slides with the secondary antibody (DyLightTM488-conjugated AffiniPure Mouse Anti-Rabbit IgG, Jackson ImmunoResearch, LABORATORIES,INC. ) diluted in PBS at a recommended ratio of 1:800. Then mount coverslip with a drop of 50% glycerol after three times wash in PBS. For confocal microscopy, a confocal scanning laser microscope (FV500, Olympus) was used. Immunofluorescence intensity was quantified with Image J (National Institutes of Health).

### 7. Statistical analysis

All data were presented as means ± S.E.M Statistically significant differences were assessed by analysis of variance (one-way ANOVA). Comparisons between two groups involved use of the Student’s t test. All statistical analysis was performed using a commercially available statistical package (SPSS 17.0). P value <0.05 denotes statistical significance.

## Results

### 1. Plasma nesfatin-1 levels

**Figure 1:**
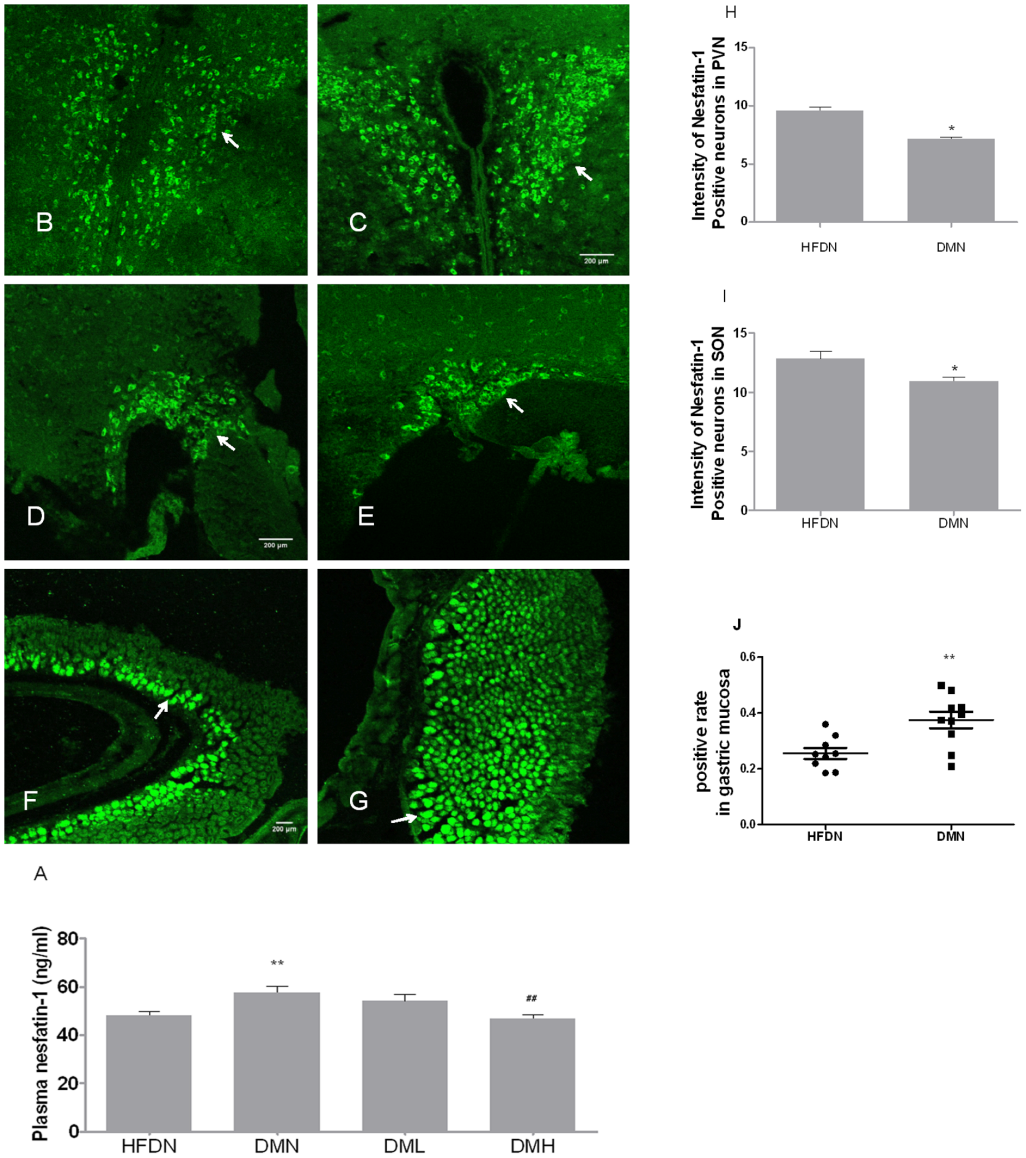
Concentration and distribution of nesfatin-1 in plasma, PVN, SON and gastric mucosa. (A) Concentrations of plasma nesfatin-1 2h after i.v. nesfatin-1 or NS injection. Nesfatin-1 expression in (B) PVN, (D) SON and (F) gastric mucosa in HFDN control mice. Nesfatin-1 expression in (C) PVN, (E) SON and (G) gastric mucosa in T2DM mice. The intensity of nesfatin-1 positive neurons in (H) PVN and (I) SON.The arrow points to nesfatin-1 positive cells. Scale bar in B, D and F = 200 µm for B-C, D-E, F-G respectively. Data were presented as means± S.E.M. *p<0.05, **p<0.01(compared with HFDN group), ^##^p<0.01(compared with DMN group).

Plasma nesfatin-1 concentrations was significantly elevated in DMN group compared with in HFDN group (Fig.1(A)). Treatment with nesfatin-1 for 6-day normalized plasma nesfatin-1 concentrations both in DML (1 µg/Kg) group and DMH (10 µg/Kg) group.

### 2. Nesfatin-1 immunoreactive cells in hypothalamus and gastric mucosa

Immunofluorescence showed that nesfatin-1 immunopositive neurons were deficient both in PVN (Fig.1(B,C,H)) and SON (Fig.1(D,E,I)) in hypothalamus. However, the increased nesfatin-1 immunopositive cells were observed in gastric mucosa(Fig.1(F,G,J)).

### 3. Blood glucose, insulin levels and insulin resistance coefficient

**Figure 2:**
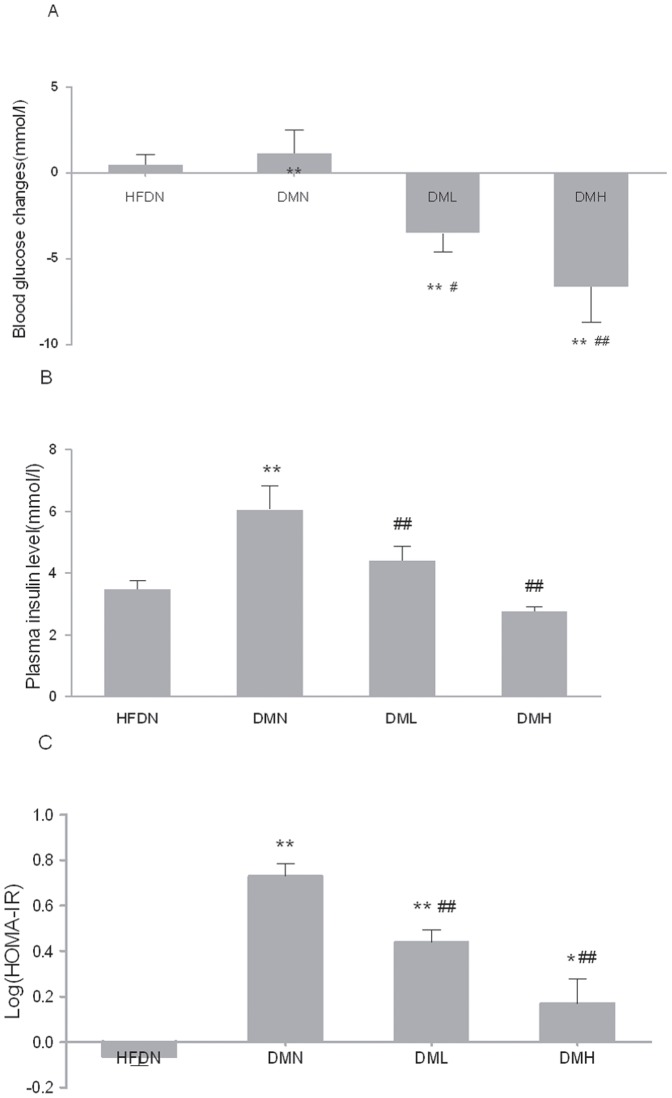
Effects of nesfatin-1 on blood glucose, plasma insulin and IR coefficient. (A) The blood glucose changes pre- and post -i.v. injection. (B) The plasma insulin levels. (C) The insulin-resistance coefficient. HOMA IR = fasting plasma insulin (µU/ml)×fasting plasma glucose (mmol/l)/22.5. All data were expressed as the mean ± S.E.M of 4–8 mice/group. *p<0.05, **p<0.01 (compared with the HFDN group). ^#^p<0.05, ^##^p<0.01 (compared with DMN group), ^+^p<0.05 (compared with DML group)

The i.v. administration of nesfatin-1 (1 µg/Kg or 10 µg/Kg) significantly reduced blood glucose in freely fed DML and DMH groups compared with HFDN and DMN groups, which was in a dose-dependent manner (Fig.2(A)).

Interestingly, the change in plasma insulin concentration was similar to that in blood glucose (Fig.2(B)). Plasma insulin levels were significantly higher in DMN than in HFDN. Insulin concentration in DML and DMH were significantly depressed with the treatment of nesfatin-1 for 6 days (1 µg/Kg, 10 µg/Kg) contrasted with DMN. These changes were significant (P<0.01). There were no significant alteration between HFDN, DML and DMH groups (p>0.05).

Insulin resistance coefficient was calculated with the formulation mentioned above. As expected, insulin resistance coefficient developed in DMN mice (Fig.2(C)) while nesfatin-1 treatment enhanced insulin sensitivity in DML and DMH significantly compared with DMN group. It is particularly of interest to note that insulin resistance was more improved in DMH group. However, insulin resistance coefficient was not normalized in both groups treated with nesfatin-1 compared with HFDN group.

### 4. Food intake and changes in body weight

**Figure 3:**
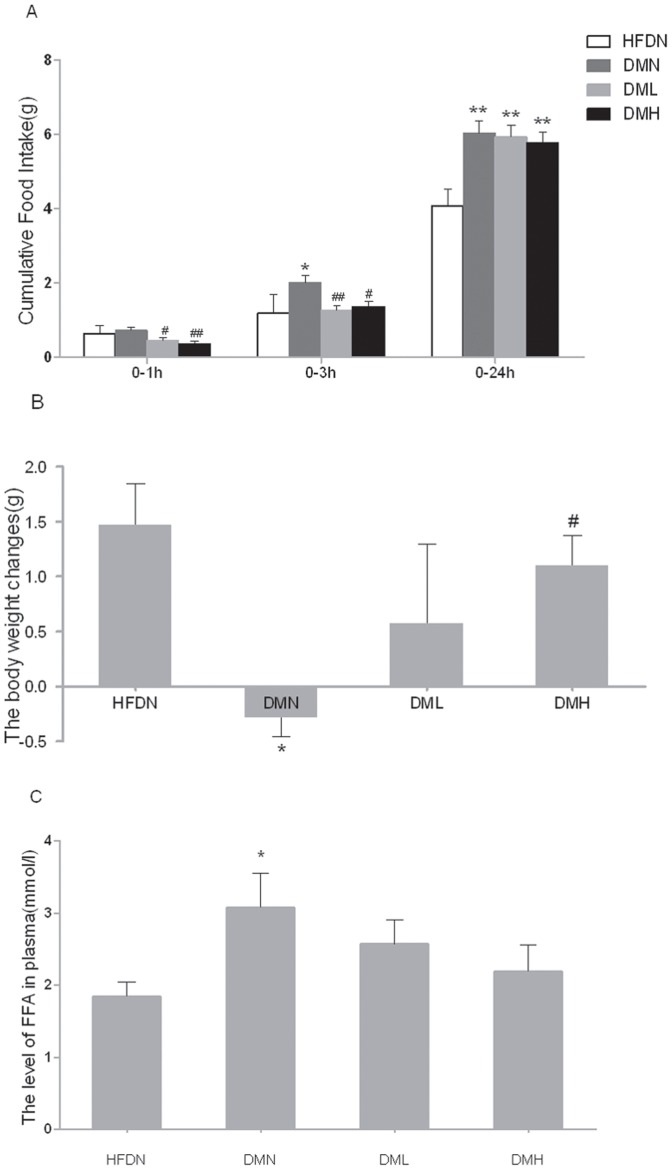
Effects of nesfatin-1 on cumulative food intake, body weight changes and FFA levels. (A) Cumulative food intake levels of 0–1h, 0–2h, 0–24h. (B) The body weight changes pre- and post- the 6-day long i.v. injection. (C) The plasma FFA levels after 6-day i.v. injection of nesfatin-1 or NS. All data were expressed as the mean ± S.E.M of 4–8 mice/group. *p<0.05, **p<0.01 (compared with the HFDN group) ^#^p<0.05, ^##^p<0.01(compared with DMN group)

Nesfatin-1 injected i.v. at the beginning of dark phase induced a dose-related decrease in food intake in the first hour (Fig.3(A)). After 3-hour injection, there still exists the significance. By contrast, the 24-hour value was not different in nesfatin-1 treated groups compared to vehicle-treated mice in DMN group.

The mean changes in body weight were remarkly decreased in DMN mice compared with HFDN mice (Fig.3(B)). Besides, treatment with nesfatin-1 for 6 days elevated the changes of body weight significantly in DMH mice. No significance was observed between DML and DMN mice (p>0.05).

### 5. Plasma FFA concentration

It turned out that FFA concentration was augmented in DMN mice than in HFDN mice (Fig.3(C)). After the treatment with nesfatin-1, FFA concentrations were normalized (p>0.05). Though difference between DML, DMH and DMN groups did not reach statistical significance, nesfatin-1 injected mice showed a trend toward a dose-related decrease in plasma FFA levels (DML 2.57±0.34 mmol/L vs. DMN 3.08±0.47 mmol/L, p = 0.31; DMH 2.19±0.37 mmol/L vs. DMN 3.08±0.47 mmol/L, p = 0.10 ).

### 6. AMPK and ACC in skeletal muscle

**Figure 4:**
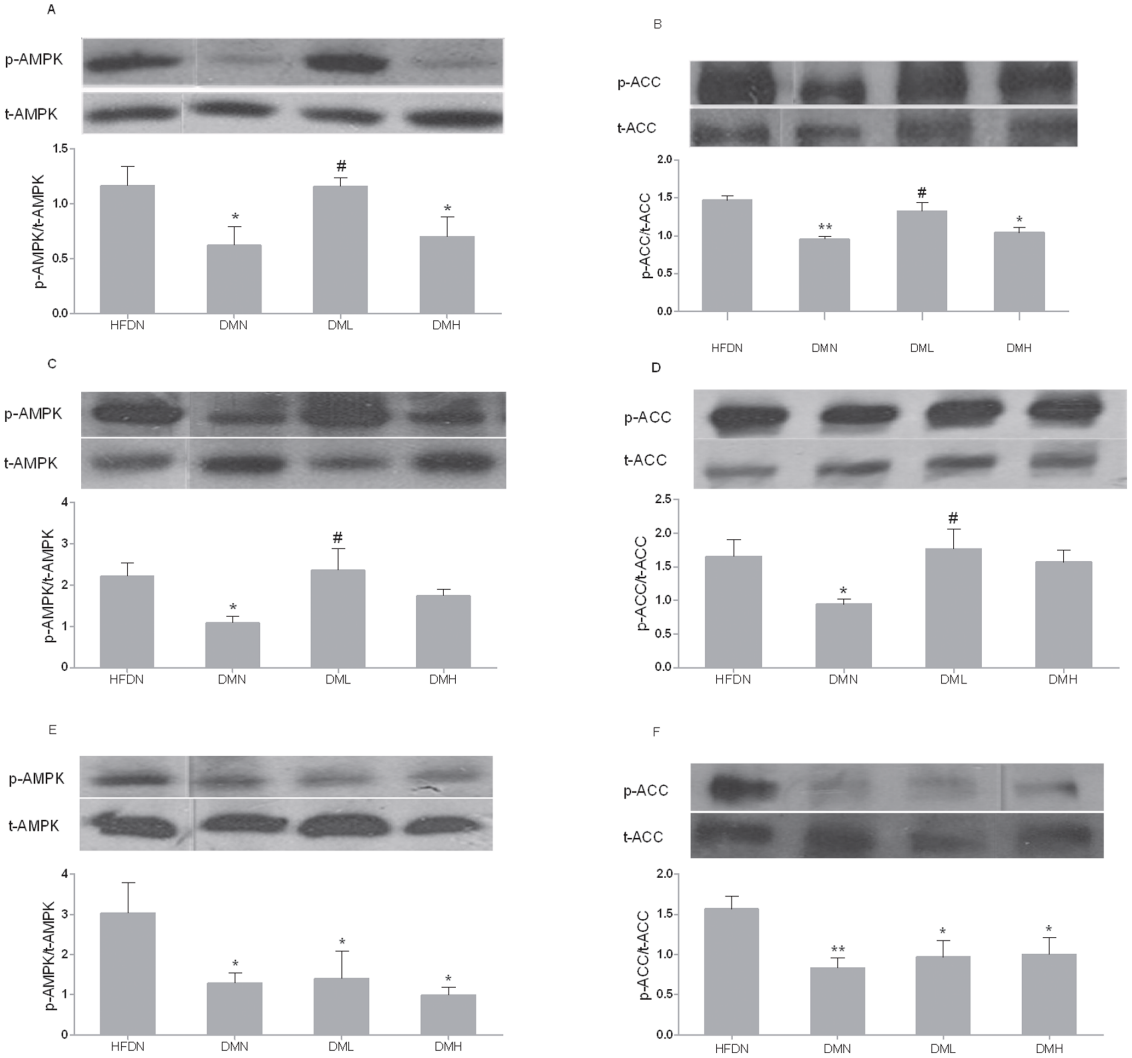
Nesfatin-1 effects on the activation of AMPK and ACC in skeletal muscle. P-AMPK relative to t-AMPK in (A) red gastroenemius, (C) soleus and (E) white gastroenemius. P-ACC relative to total t-ACC in (B) red gastroenemius (D) soleus and (F) white gastroenemius. All data were expressed as the mean ± S.E.M of 4–8 mice/group. *p<0.05, **p<0.01 (compared with the HFDN group)^ #^p<0.05 (compared with the DMN group)

In type 2 diabetic mice, our results showed that the phosphorylation and activation of α-AMPK and β-ACC was dysregulated (Fig.4). The phosphorylated AMPK and ACC expression was upregulated compared with DMN group( Fig.4 (A-D)) when treated with low dose nesfatin-1 (1 µg/Kg, i.v.). There is no significant alteration between DMH and DMN mice.

The effect of i.v. nesfatin-1 on the phosphorylation of α-AMPK and β-ACC was more pronounced in red (slow twitch, oxidative) skeletal muscles, which have higher rates of fatty-acid oxidation, than in white (fast twitch, glycolytic) muscles. In gastrocnemius white muscle, there was, however, no significant difference between the DMN group and the nesfatin-1 treated groups (see Fig.4( E, F).

## Discussion

Type 2 diabetic mice induced by a high-calorie diet and two low-dose STZ injections were widely accepted in diabetes research field [19]–[21]. The blood glucose and the plasma insulin levels were high, which fitted for the clinical phenomena. The food intake and serum FFA levels were increased while the body weigh gain was decreased, all of which are consistent with characteristics of type 2 diabetes [19], [21], [25]. The diabetic mice also expressed a decreased level of p-AMPK and p-ACC, which displayed the abnormality of fatty acid metabolism [26]. Therefore, the type 2 diabetic mice could be used to study the fat metabolism disorders and the distribution of nesfatin-1.

We have investigated the central and peripheral distribution of nesfatin-1 in T2DM and demonstrated a novel function of peripheral nesfatin-1 in regulating fatty acid metabolism. It was the first time to report increased expression of nesfatin-1 in gastric mucosa and decreased expression in hypothalamic nucleus in type 2 diabetic mice induced by a combination of a high-calorie diet and two low-dose STZ injections.

We speculated the increased plasma nesfatin-1 was secreted from gastric mucosa and this change was originated from the reduced central nesfatin-1. Increased peripheral nesfatin-1 acted as a compensatory role, thus promoting nutrient balance. Interestingly, chronic intravenous administration of nesfatin-1 (6-day) normalized the increased plasma nesfatin-1 levels in diabetic mice. Plasma nesfatin-1 we measured 2h later of the last administration was almost from the endogenous source, since the half-life period of nesfatin-1 is short (9–10 minutes) [3], . The normalization of nesfatin-1 in diabetic mice might be a result of sufficient effect of exogenous nesfatin-1.

The normalized insulin resistance combined with the normalized plasma FFA levels and body weight changes indicated a sufficient utilization of fatty acid and an improved fatty acid metabolism in skeletal muscle. This view was strongly accepted by research of this field [27]. To study the downstream signaling pathway, we detected the AMPK and ACC activation. AMPK functions as a cellular fuel regulator, senses the whole-body energy balance and regulates the insulin resistance and fat metabolism [17], [26], [28]. P-AMPK phosphorylated ACC, thus ameliorated the malonyl CoA levels and then decreased the inhibition to CPT-1 and fat synthesis. In skeletal muscle, the actions of AMPK and ACC were thought to be mediated mainly through the α-AMPK and β-ACC isoform respectively [14], [17] The expression and activities of α-AMPK were dysregulated in high-fat feeding rats, while activating α-AMPK by metformin obviously ameliorated high-fat induced insulin resistance [17], [18]. In our study, there was increased expression of phosphorylated AMPK and ACC in soleus and red gastrocnemius muscle (slow twitch, oxidative) of the low dose nesfatin-1 treatment. But it stayed unchanged in the white gastrocnemius muscle (fast twitch, glycolytic) [14]. Low dose i.v. injection of nesfatin-1 regulated fatty acid metabolism in muscles with activation of AMPK-ACC pathway, at least partly. Interestingly, the high-dose administration of nesfatin-1, which decreased the related whole-body index, has not altered the AMPK-ACC link. We presumed that the change in p-AMPK and p-ACC was caused by the receptor desensitization while the dose-dependent change in some index such as glucose and insulin levels were caused by the long-term buildup effect of nesfatin-1. It was reported that nesfatin-1 receptor in the hypothalamus was G protein-coupled receptor and several regulatory peptides with G protein-coupled receptor-mediated actions, including ghrelin, have been shown to cause rapid receptor desensitization [29], [30].

**Figure 5:**
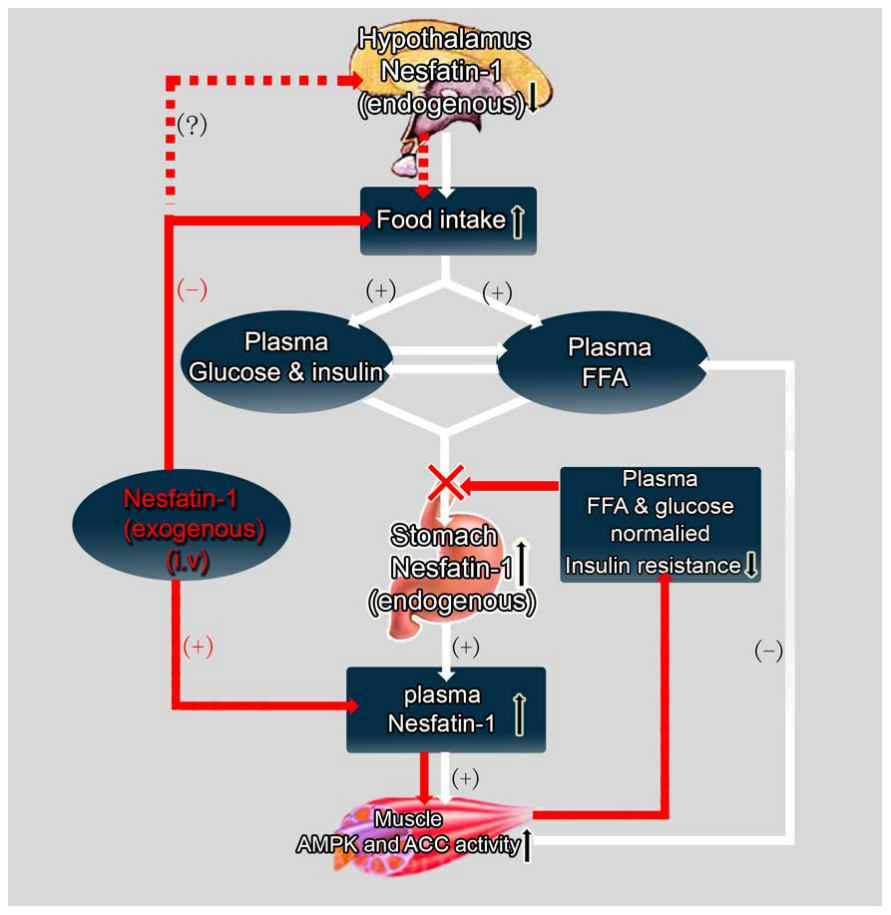
Effect of nesfatin-1(both endogenous and exogenous) on fat metabolism in the muscle in STZ-treated mice. The pathway of endogenous nesfatin-1 effect is showed by the white arrow and that of the exogenous nesfatin-1 is showed by the red arrow. The dashed arrow shows the possible pathway of exogenous nesfatin-1 affecting food intake.

In conclusion, our results indicated that the decreased nesfatin-1 expression in hypothalamus contributed to diabetic hyperphagia in the early stage during the development of type 2 diabetes, while peripheral nesfatin-1 functioned chiefly to regulate energy metabolism. The role of nesfatin-1 in diabetes is indicated in figure 5. The excessive energy intake results in heavy metabolic burden of body, so the plasma FFA, glucose are elevated. Increased expression of nesfatin-1 in stomach aims to improve energy conditions. However, the endogenous nesfatin-1 is not sufficient for the normalization of FFA and glucose et al. Supplementary exogenous nesfatin-1 normalized the level of insulin, decreased blood glucose and improved insulin resistance, all those changes are partially attributed to promoted FFA metabolism. Improved energy condition could inhibit the compensatory overexpression of nesfatin-1 in gastric mucosa. Our study tended to fulfill the blank of whether nesfatin-1 could regulate the fat metabolism in the T2DM mice. We will further study the desensitization of nesfatin-1 receptor and if the central nesfatin-1 has an effect on lipid metabolism.
